# 657. Skin Grafting Approaches and Outcomes for Reconstructive Pediatric Burn Surgeries: A NSQIP-P Study

**DOI:** 10.1093/jbcr/irag033.494

**Published:** 2026-04-06

**Authors:** Angad S Sidhu, Nikola Vuckovic, Alexander Smith, George Corpuz, Leigh J Spera

**Affiliations:** Indiana University School of Medicine, Indianapolis, IN; Indiana University School of Medicine, Indianapolis, IN; Indiana University School of Medicine, Indianapolis, IN; Indiana University School of Medicine, Indianapolis, IN; Indiana University/Eskenazi Health, Indianapolis, IN

## Abstract

**Introduction:**

Pediatric burn injuries remain a leading cause of trauma-related morbidity worldwide, often necessitating surgical reconstruction to restore function and minimize disability. Skin grafting is the most employed reconstructive technique, yet most data on pediatric surgical burn management are derived from single-center series. Consequently, predictors for grafting approaches and short-term outcomes remain poorly defined in large, heterogenous pediatric populations. This study utilizes the ACS-NSQIP-P database to characterize national patterns and associated factors involved in skin grafting and their outcomes for pediatric patients undergoing burn reconstruction.

**Methods:**

The NSQIP Pediatric database (2018–2023) was queried for patients <18 years undergoing surgical treatment of burns. Procedures were grouped as split-thickness skin graft (STSG), full-thickness graft, skin flap, or tissue expander. Multivariable logistic regression was performed using IBM SPSS Statistics (v31.0) to identify independent predictors of 1) STSG versus other reconstruction types and 2) minor and major 30-day postoperative medical and surgical complications including but not limited to readmission, return to operating room, and/or mortality. Odds ratios (OR) with 95% confidence intervals (CI) were reported. *p*-values <0.05 were statistically significant.

**Results:**

A total of 1575 patients were included (62.5% male, median age 8 years). STSG was the most common operation (82%). On multivariable analysis, STSG was less likely associated with outpatient status (OR 0.16, 95% CI 0.08–0.34, *p*<.001), first-degree burns (OR 0.03, 95% CI 0.01–0.14, *p*<.001), or upper extremity location (OR 0.15, 95% CI 0.05–0.42, *p*<.001), and more likely with ASA III/IV (OR 6.05, 95% CI 1.85–19.9, *p*=.003). Complications occurred in 9.4% of patients, most commonly following trunk burns (26%). Independent predictors of complications included trunk location (OR 5.02, 95% CI 1.14–22.1, *p*=.033), longer operative time (OR 2.33 per SD, 95% CI 1.72–3.16, *p*<.001), Hispanic ethnicity (OR 3.77, 95% CI 1.91–7.43, *p*<.001), and ASA III/IV (OR 2.86, 95% CI 1.22–6.68, *p*=.015).

**Conclusions:**

In this national cohort, split-thickness grafting was the predominant treatment for pediatric burns. Surgical decision-making was driven primarily by burn depth, location, and patient acuity, while complications were associated with trunk involvement, longer operative times, and higher ASA status. These findings provide contemporary benchmarks for outcomes in pediatric burn surgery and highlight targets for perioperative optimization.

**Applicability of Research to Practice:**

This study provides national benchmarks for pediatric burn reconstruction and identifies patient and operative factors that influence surgical decision-making and short-term complications. These findings can inform preoperative counseling, perioperative optimization, and tailor postoperative monitoring.

**Funding for the study:**

N/A.

